# Supplementary material for: Quantitative X-ray phase-contrast microtomography from a compact laser-driven betatron source
Source: Nat Commun. 2015 Jul 20;6:7568. doi: 10.1038/ncomms8568 (PMC4518247; doi:10.1038/ncomms8568)
Supplement: Supplementary Information — Supplementary Figures 1-5, Supplementary Note 1 and Supplementary References. [file ncomms8568-s1.pdf]

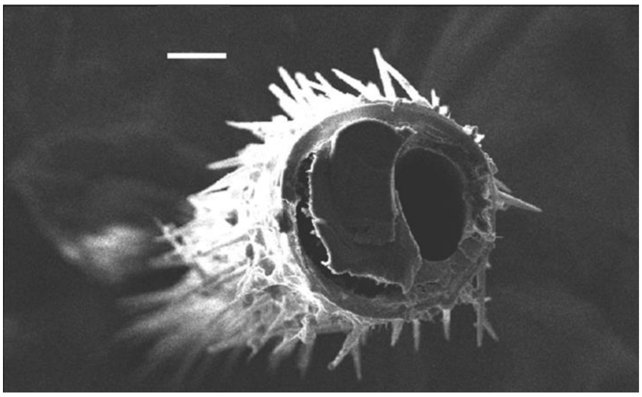

Supplementary Figure 1: Scanning electron microscope image of a cross section trough a leg of the dried sample used in the experiments. White scale bar corresponds to 20 μm.

Since a cuticle of such an insect is mainly composed of chitin fiber structures with a density of 1.6 g cm^{-3} (see Fig. 7 in [1]), we can model it by a doughnut shape with the wall electron density of $5.2x10^{23}$ cm^{-3}. (Assuming pure chitin ($C_8H_{13}NO_5$) with a density value of 1.61 g cm^{-3}).

The object of comparison was selected to be a leg - for easier identification distant from any other parts - as indicated by the red arrow in Supplementary Fig. 2. Its outer diameter according to an optical microscope image is approx.140-160 μm (see Supplementary Fig. 2).

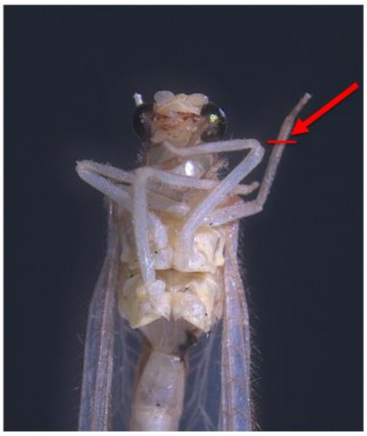

Supplementary Figure 2: **Microscope image of the sample**. The red arrow indicates the leg of the object that was used for the comparison. The outer diameter is according to an optical microscope image approx.140-160 μm.

Although the single shot X-ray images have a pixel resolution of 6 μm (as stated in the main text of the manuscript) the tomogram will suffer additional blurring due to reconstruction method and the angle step size of the data set. We model the overall loss in resolution by introducing a point-spread function (PSF) of the measurement. Its convolution with the ideal object would than resemble our tomographic measurement.

Assuming the PSF to be a 2D Gaussian function in the form of

$$f\left(x,y\right)=A\cdot\exp\left(-\frac{x^2}{2\sigma^2}-\frac{y^2}{2\sigma^2}\right), (1)$$

(as shown in Supplementary Fig. 3c) the convolution will result in a blurred image with consequently reduced maximum signal for structures smaller or comparable to the PSF.

For 2D Gauss PSF with a FWHM of 36 μm the maximum reconstructed density for our test object (insect leg, outer diameter about 150 μm and inner diameter about 125 μm) will drop to 33% of its absolute value (see Supplementary Fig. 3b).

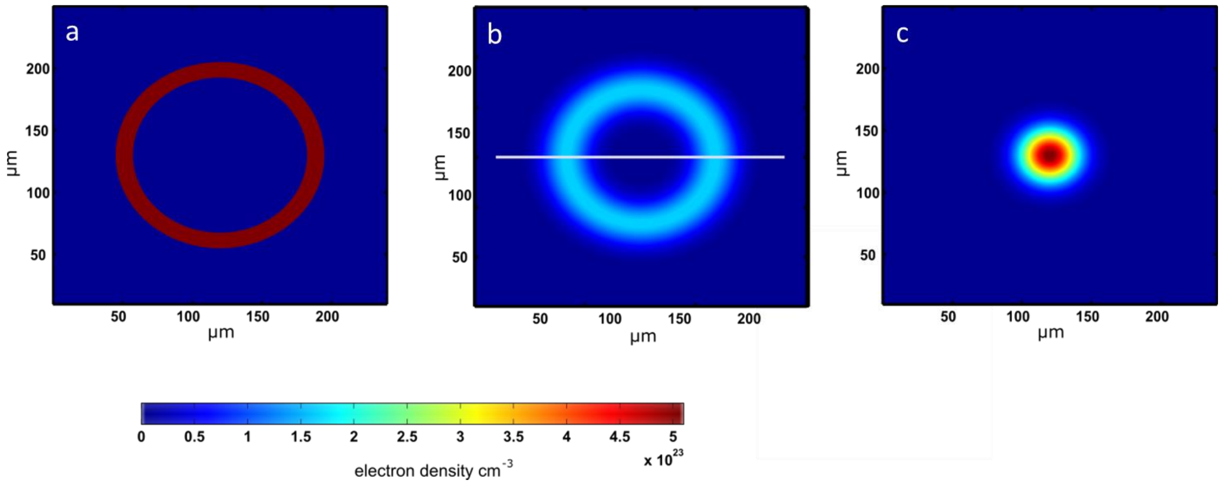

Supplementary Figure 3: **Model of the Point Spread Function. a:** Doughnut shape with a step like molecular chitin electron density of $5.2x10^{23}$ cm^{-3}, an outer and an inner diameter of 150 μm and 125 μm. **b**: Convolution of the test object (Fig. 3a) and a Gaussian PSF (Fig. 3c). A lineout of it (white) is shown in the Supplementary Fig. 5. The color map applies for the Fig. 3 a and b . **c**: 2D Gauss PSF with a FWHM of 42 μm.

The estimate for the FWHM of PSF can be obtained by evaluating the reconstruction of the originally sharp feature. We used the edge of the needle - the holder of our sample - whose blurring effect appears to be 3-4 reconstruction voxels, corresponding to 36 μm - 48 μm.

Returning to our test object one can perform a comparative analysis between the lineout through our convolutional model (white line in the Supplementary Fig. 3b) and a lineout in the corresponding slice within the tomogram (indicated by the red line in Supplementary Fig. 4b). Supplementary Fig. 5 shows this comparison for PSF widths of 24, 36, and 48 μm FWHM, i.e. 2,3,4 reconstruction voxels.

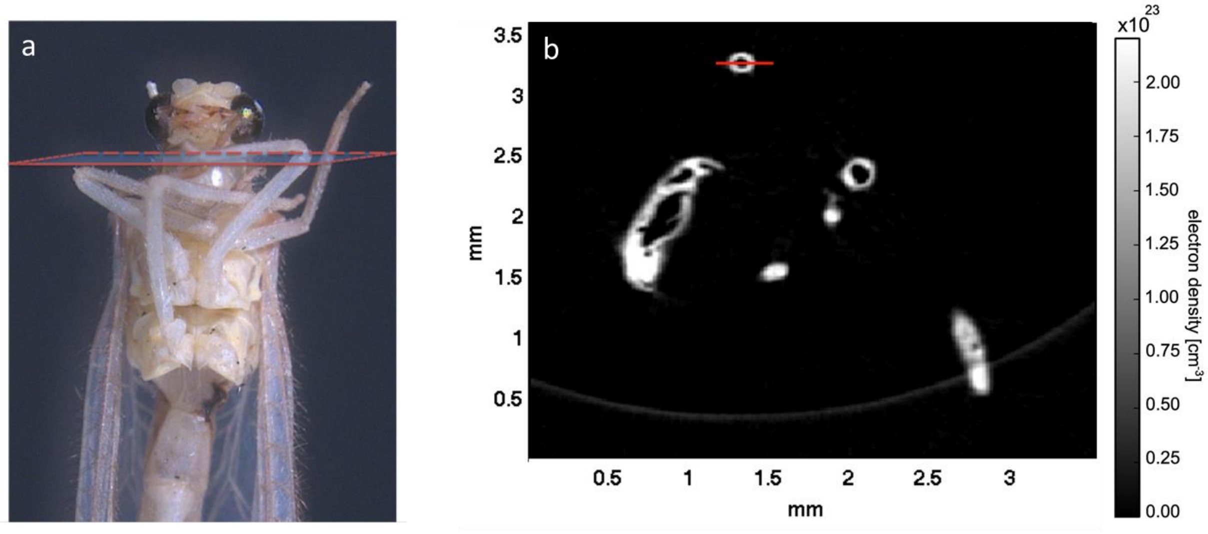

Figure 4: **a**: optical microscope photograph of the object with the red-rimmed plane indicating the position of the reconstructed slice on the right. **b**: Reconstructed slice through the object. The lineout of a leg (red) is plotted in Supplementary Fig. 5.

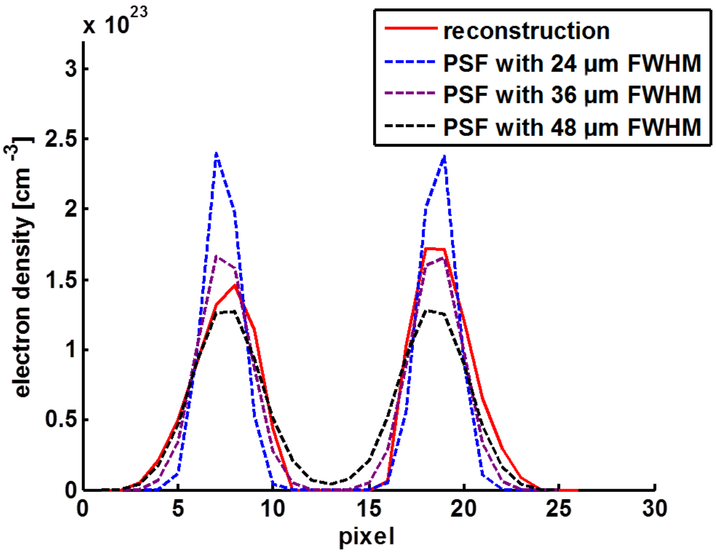

Supplementary Figure 5.: Comparison of the reconstructed lineout and modeled lineout for a Gaussian PSF with 24, 36, 48 µm FWHM and the model being a doughnut with a step like molecular chitin density profile of 5.2×10^{23} cm^{-3} and an outer and inner diameter of 150 µm and 125 µm, respectively.

The reconstructed electron density values agree well with the model for a PSF FWHM approx. 36-48 µm, corresponding to the estimated 3-4 reconstruction voxels. This fact explains the reduction of reconstructed electron densities within the small features of the insect compared to the molecular chitin. Taking into the account the technical limitation of the method the reconstructed values are in good agreement with correspondingly expected absolute electron density values.

Supplementary Note 1

On the absolute electron density.In order to strengthen the attribute ‘quantitative’ in our title we have compared the reconstructed electron densities within an insect’s leg with a model imitating the structure and density of such profile processed according to imaging and reconstruction properties of our setup.

An insect leg was chosen due to the simplicity of its geometry (a cylindrical shape) and the type of its composition material (mainly chitin). From a scanning electron microscope image of a cross section (of the insect’s leg used in the experiments, see Supplementary Fig. 1) it can be seen that most of the inner part is void (due to vacuum desiccation). Furthermore the wall thickness of the cuticle varies between 9-14 □m.

References

1. Vincent, J. F. V & Wegst, U. G. K. Design and mechanical properties of insect cuticle. *Arthropod Struct. Dev.* **33,** 187–199 (2004).
